# Salmagundi

**Published:** 1886-05

**Authors:** 


### SALMAGUNDI.

EDITED BY E. G. BETTY, D.D.S.

CHICAGO converted Sam. Jones from chewing tobacco and Sam. Small from smoking cigarettes.

CHEAP though pure chloroform is now made by a somewhat new process in which alcohol is not employed.

COCAINE was discovered by Senor Pizzi in 1857 " in the humble laboratory of la Boticany Drogurria Boliviana."

DURING the year 1885 there occurred in England twelve deaths from chloroform narcosis and three from ether.

M. Pasteur will soon be covered with medals and orders, the last being that of the Legion of Honor, to be conferred by the French Government.

---

Dr. Werner Siemes, the great electrician, has given the German Government $125,000 for the establishing of an institute for scientific experiments.

---

Our friend, Dr. C. M. Wright, of Cincinnati, has recently purchased and fitted up a commodious house on West Seventh street for office and residence where we wish him all happiness and prosperity.

---

Two ministers of the East Pennsylvania Conference have made public confession of the use of tobacco. One of them smoked to cure toothache, and the other for a throat trouble. The Conference excused them and an epidemic of toothache among the ministers is feared.—*Boston Record.*

---

"The most complete summary of our knowledge about the therapeutic properties of cocaine is a work of over a hundred pages entitled "Coca Erythroxylon and its Derivatives," published by Parke, Davis & Co., Detroit, for gratuitous distribution to the medical profession. We advise our readers to apply for a copy of it."

---

Prof. Bonafoux, at a recent meeting of Academy of Medicine, at Paris, read a paper upon a powder which possesses great hemostatic power, and is capable, it is said, of arresting the bleeding of large arteries, so that it will prove serviceable in important surgical operations. The powder is prepared by mixing equal parts of colophony, carbon, and gum arabic.—*Medical and Surgical Reporter.*

---

The spunky wide-awake little Mad River Dental Society holds its annual meeting at the Phillips House, Dayton, O., May 18th, 1886, at 9 o'clock A.M., or as soon thereafter as possible, and continues in session one day and evening. As a member, we extend a cordial invitation to all dentists in this "neck of the woods" to shut up shop for that one day and come with us. The papers will be good and the discussions short, sharp, and decisive.

---

THE *National Druggist* says that persons who have a superstitious dread of Friday will not be pleased to learn that this is a thoroughly Friday year. It came in on a Friday, will go out on a Friday, and will have fifty-three Fridays. The are four months in the year that have five Fridays each; changes of the moon occur five times on a Friday, and the longest and shortest day of the year each falls on a Friday. Be careful that you do not sell morphine for quinine on Friday.

---

WE have read with pleasure and profit a work by the Rev. Josiah Strong on "Our Country! Its Possible Future and Its Present Crisis," and though we do not agree altogether with the author, yet we can recommend the work to the readers of the REGISTER with the assurance that they will find it a mine of statistical wealth in a nutshell. It is published by the American Home Missionary Society, Bible House, Astor Place, New York, and sells for thirty-five cents, cloth.

---

AMONGST a number of medico-legal cases reported by Henry A. Riley, Esq., of New York City, to the *Medical and Surgical Reporter*, occurs the following: "In a recent French case, a dentist was sued for damages resulting from the death of a patient through the use of an anæsthetic. The court held that the dentist was neither a physician nor a health officer, and could not legally administer the anæsthetic. He was adjudged guilty of manslaughter, but the punishment imposed was only a fine of 3,600 francs."

---

RETENTION CYST OF THE SUBMAXILLARY GLAND.—Dr. Gerster presented the specimen, which he removed from a man aged twenty-six years. The tumor had previously been taken for a ranula. Tapped through the mouth, and collapsed; but it refilled, and became so large as to interfere seriously with deglution, and even with breathing. Dr. Gerster removed it, and found it to be a retention cyst of the submaxillary gland developed toward the median line, and thus giving the impression of a dermoid cyst. The facial and lingual arteries were ligated during the operation.—*Medical and Surgical Reporter.*

---

Antiseptic Mouth-Wash.—Dr. Miller (*Duetsch. Med. Woch.*) found that, by using the following mixture, he could completely sterilize the mouth, cavities in carious teeth, etc.

| | | |
|---|---|---|
| R. | Thymol, | 4 gr. |
| | Benzoic acid, | 45 gr. |
| | Tincture of Eucalyptus, | $3\frac{1}{2}$ fl. dr. |
| | Water, | 25 fl. oz. |

The mouth is to be well rinsed with this mixture, especially just before going to bed, since most of the damage by fermentative and putrefactive processes in the mouth is done at night, during sleep, unless the exciting cause be previously removed or rendered inert.

---

One of the most delightful, charming, and fascinating recreations for both indoors and outdoors is amateur photography. It brings into play both artistic and scientific knowledge, "develops" a love for the beautiful in nature; "negatives" all that is degrading or debasing; brings to a "focus" a clear "image" in the mind of what God intended the world to be to us; "prints" it indelibly upon the "sensitive" memory, and "lens" to us a better idea of our place in creation, besides stimulating our love for our fellow-men, faith in God, hope in the future, and charity for all. If the reader would like to know all this, let him apply to our friend Dr. Frank Hunter, who has just completed a sure, quick, and warranted-not-to-fail-to-go off drop shutter.

---

A review of Prof. Oscar Schmidt's work on "The Mammalia in Their Relation to Primeval Life," says: "Prof. Schmidt asserts that man has five teeth less than he had originally, and he predicts that the intellectually higher classes of the future will have fewer teeth than are now allotted to the race. If man modifies his natural quota of teeth by culture, the more radical theory of evolution is vindicated to some extent. But that "if" has a stubborn appearance." The last sentence of the reviewer harmonizes thoroughly with the opinion of the dental world that there is as yet no reason to suppose that the third molar is gradually being obliterated for want of use and other causes. However, if there be any truth in the doctrine that when an organ is thrown out of use nature dispenses with it, the dentist of some thousands of years hence will be racking his brains to find out if man really ever possessed the much-talked-about third molar.

---

THE M. V. D. A. gold medal for the best essay is thus spoken of by Dr. Watt in an editorial in the *State Journal* for April:

We hope the offer of a gold medal for the best essay submitted, if found worthy, will call out a goodly number of instructive papers. We do not know what disposition will be made of the papers not receiving the award, but if returned to their authors, they will probably come to light through the periodicals, and the profession will get the benefit of the thoughts and suggestions they contain. Of course the authors need not tell the public, nor even the editors, their history. If an abundance and variety of thought result from the offer, it would be unfortunate to have it lost to the profession. We hope there will be lively competition for the award among the talented and ambitious young brethren.

This offer of a medal is in accord with former action of the Association. At its meeting in 1855 a prize of one hundred dollars was given for a popular essay, and a fifty dollar prize was given for a set of extracting instruments, the Association keeping the copyright of the essay and returning the instruments to the manufacturer.

At a later date it offered a large prize for an essay on alveolar abscess, but no award was made, as the special committee did not regard any one among the papers offered as worthy of it. In addition to these, two members of the profession, Dr. S. S. White and W. H. Morgan, offered a prize of two hundred dollars for an essay on The Health of Dentists, the examination and award to be made by the Association, in accordance with the precedents it had established; but no award was made on this subject, for reasons similar to those governing in the alveolar abscess offer.

At the meeting in which the DENTAL REGISTER was transferred to Dr. James Taylor, the Association resolved that it would, hereafter, use a liberal proportion of its funds in the way of awards and prizes, in order to stimulate the energies of those competent, so as to increase the rate of professional progress. Hence this proposed award is in consonance with both precedent and resolution, in a line that has probably resulted in profit to the profession and the public.

